# Maternal Mental Health Disorders and Reports to Child Protective Services: A Birth Cohort Study

**DOI:** 10.3390/ijerph14111320

**Published:** 2017-10-30

**Authors:** Ivy Hammond, Andrea Lane Eastman, John M. Leventhal, Emily Putnam-Hornstein

**Affiliations:** 1 Children’s Data Network, Suzanne Dworak-Peck School of Social Work, University of Southern California, Los Angeles, CA 90089, USA; ihammond@usc.edu (I.H.); andrea.eastman@usc.edu (A.L.E.); 2 School of Medicine, Yale University, New Haven, CT 06510, USA; john.leventhal@yale.edu; 3 California Child Welfare Indicators Project, University of California at Berkeley, Berkeley, CA 94720, USA

**Keywords:** mental health disorders, pregnancy, infant health, child protective services, child maltreatment

## Abstract

*Background*. Existing literature has documented a strong relationship between parental mental illness and child maltreatment, but little is known about the prevalence of mental illness among childbearing women. In the present study, linked administrative records were used to identify the prevalence of maternal mental health (MH) disorders documented at birth and determine the associated likelihood of maltreatment reports during infancy. *Materials and Methods*. Vital records for California’s 2006 birth cohort were linked to hospital discharge and Child Protective Services (CPS) records. The International Classification of Diseases, Ninth Revision, Clinical Modification (ICD-9-CM) billing codes from the mother’s delivery hospitalization were used to determine diagnosed maternal MH disorders for 551,232 infants born in 2006, and reports of alleged maltreatment were documented from CPS records. Vital birth records were used to control for sociodemographic factors. Finally, the associated risk of reported maltreatment during the first year of life was examined using generalized linear models. *Results*. Among infants in this statewide birth cohort, 2.8% were born to a mother with a documented MH disorder, of which 41.3% had documented maternal substance abuse issues versus less than 0.5% of infants born to mothers without a diagnosed MH disorder. Further, 34.6% of infants born to mothers with a MH disorder were reported to CPS within one year, and a majority of those reports were made within the first month of life (77.2%). In contrast, among children born to mothers without a MH disorder, 4.4% were reported to CPS during infancy. After controlling for sociodemographic factors, the rate of CPS reports during infancy for infants born to mothers with a MH disorder but no substance use disorder was 2.6 times that of infants born to mothers without a MH disorder (95% CI = 2.47, 2.73). Among infants born to mothers with MH and substance use disorders, the rate of CPS reports during infancy was 5.69 times that of infants born to mothers without a MH disorder (95% CI = 5.51, 5.87). *Conclusions*. Administrative records provide a method for identifying infants born to mothers with MH disorders, enabling researchers to track rates over time and generate population-level data to inform policy development and improve service delivery.

## 1. Introduction

The movement to deinstitutionalize psychiatric care in the 1970s led to notable increases in pregnancy and childbirth among women with diagnosed mental health (MH) disorders [1]. Whereas many mothers with MH disorders give birth to healthy infants and demonstrate appropriate parenting practices, existing research indicates that children of mothers with a history of mental illness are at heightened risk of adverse health and wellbeing outcomes, including birth abnormalities [2], low birth weight [3], preterm delivery [4,5,6], insecure parent-child bonding [7], and the transmission of mental illness [8]. Empirical literature has also documented a strong relationship between parental mental illness and child abuse and neglect [8], particularly among children living with a mother with mental illness [9,10] and within economically disadvantaged families [9].

Given the broad range of functional impairments and severity in symptoms across MH disorders [11,12,13], the effect of these disorders on parenting capacity and child safety almost certainly varies. One study found that Child Protective Services (CPS) reports were more likely for children of mothers with mental illness than for children born to mothers without mental illness, regardless of the type of diagnosis [12]. Another study found that mothers living with depression were less likely to use recommended infant care practices if they also had been diagnosed with a personality disorder [14]. Because researchers attempting to account for this heterogeneity have categorized MH disorders in different ways, corroborating findings across studies is difficult [13,14,15]. As such, prevalence estimates for specific mental disorders among mothers are not well-documented, and the relationships between the various categories of MH disorders and children’s outcomes, including involvement with CPS, remain largely unknown.

The International Classification of Diseases, Ninth Revision, Clinical Modification (ICD-9-CM) codes offer a standardized method for tracking population-level changes in diagnosis rates over time and have previously been used to document the prevalence of maternal MH and substance-related disorders [16,17] and their association with subsequent maltreatment and CPS involvement [12,15,18]. No studies, however, have assessed the occurrence of a CPS report regarding infants born to mothers with a MH disorder known to medical professionals at the time of delivery, which is a critical engagement and intervention point. In the current study, linked administrative records from California’s 2006 birth cohort were used to determine the following: (1) the prevalence of infants born to mothers with a MH disorder documented in their medical records at the time of delivery, stratified by disorder type; (2) the cumulative percentage of infants reported to CPS during the first 12 months of life among those born to mothers with a MH disorder; and (3) the risk of a report to CPS in those infants born to mothers with a diagnosed MH disorder versus other infants after adjusting for co-documented substance exposure and other factors.

## 2. Materials and Methods

### 2.1. Data Sources

The analytic dataset was constructed by linking three administrative data sources from California: (1) vital birth records for all infants born in 2006; (2) maternal hospital discharge records corresponding to those births; and (3) CPS records for allegations of abuse or neglect made within 12 months of birth. The authors probabilistically linked birth records to CPS records using available unique (i.e., maternal Social Security number) and non-unique (e.g., parent and child first names or parent and child dates of birth) personal identifiers. Among infants reported to CPS and born in 2006, 92% were successfully linked to a birth record. In a separate linkage, 97% of maternal hospital discharge records were probabilistically linked to birth records as part of the ongoing California Vital Stats to Care Delivery Project [19]. The two linked files were then de-identified and merged using the unique birth-record number common to both files. The analysis includes an estimated 95% of all live births in California during 2006, excluding births that occurred in military hospitals and at home. These linked data were previously used to evaluate CPS reporting among mothers with a history of documented substance abuse [20]. This study received human subject approvals from both state (CA HHS #09-12-51) and university (UCB #2010-01-592) institutional review boards and was additionally reviewed by all departments from which data were obtained.

### 2.2. Variables

#### 2.2.1. Dependent Variable

The dependent variable was defined as a first report of alleged maltreatment made to CPS during the first 12 months of life. All first reports of maltreatment were included, irrespective of whether the allegation was investigated or substantiated by CPS. Existing research has documented that infants reported to CPS are at heightened risk of negative outcomes, including re-reporting to CPS, placement into foster care, and critical injury and fatality, regardless of whether or not the allegations were substantiated [21,22]. The decision to include all allegations arose from an interest in clinical and community decisions to report infants born to mothers with MH disorders, as opposed to the type of response to those reports by the CPS system.

#### 2.2.2. Independent Variables

Maternal MH disorders were coded based on ICD-9-CM diagnostic codes [23]. Up to 25 codes were documented in the medical records of delivery hospitalizations. Each birth was categorized based on the presence of an MH disorder documented at the time of birth (i.e., no MH disorder or MH disorder). Subsequently, births with a MH disorder were grouped into four non-mutually exclusive diagnostic categories used in previous research: (1) psychotic disorders; (2) mood disorders; (3) anxiety disorders; and (4) MH Disorder at Delivery [12,15,17]. Each category was dichotomously coded, and mothers with more than one diagnosis fell into multiple categories. Mental disorders at delivery is an ICD diagnostic code (648.4) used to document any mental disorder complicating pregnancy, childbirth, or the puerperium [16]. We excluded disorders occurring in childhood, personality disorders, and mental disorders attributable to medical conditions in an effort to focus on the relationship between a mother’s experience of a MH disorder leading up to childbirth and CPS involvement for the infant. Our decision was informed by parameters established in earlier studies that used the same data sources from California [16,17]. A full list of included codes and the corresponding number of births for each are included as Appendix A.

#### 2.2.3. Covariates

To characterize mothers diagnosed with a MH disorder and isolate the association between a diagnosed maternal MH disorder and CPS reports during infancy, several covariates available in the birth records were examined. Covariates included maternal race/ethnicity (White, Black, Hispanic, or other/missing) and maternal age at first-ever birth in years (≤19, 20–24, 25–29, and ≥30). The decision to use maternal age at first-ever birth aligns with research indicating that the timing of a first birth is an important proxy for future outcomes of a mother and her children [24,25]. Insurance type was coded dichotomously (private or public). California allows for retroactive enrollment in the state’s public system if there is no insurance coverage at the time of birth. Additional covariates included paternity establishment (established or missing), the timing of the initiation of prenatal care (first trimester, second trimester, and third trimester/no care), and parity (first birth or non-first birth). Finally, all births were coded based on the presence or absence of an ICD-9-CM code in the mother’s or infant’s hospital discharge records indicating maternal substance use or prenatal substance exposure (no or yes)*.* A list of ICD-9-CM substance exposure codes is also included in Appendix A.

### 2.3. Analysis

Covariate distributions were assessed using χ^2^ tests to determine statistically significant variations (*p* < 0.05) based on a diagnosed MH disorder. Distributions of covariates were also assessed for specific MH diagnostic categories. The relationship between a diagnosed maternal MH disorder and CPS reporting was descriptively presented as the cumulative percentage of infants reported to CPS by month throughout the first year of life. To isolate the increased risk of CPS reporting associated with a diagnosed MH, a series of generalized linear models were estimated based on a Poisson distribution, log link, and robust standard error adjustments to account for binary outcomes [26,27]. Estimates were reported as risk ratios (RR) with 95% confidence intervals (95% CI) and presented for any MH disorder, as well as specific categories of diagnosed MH disorders. Model 1 shows the unadjusted risk of being reported for an infant born to a mother with a MH disorder. Model 2 reflects the main effects of MH disorders and includes interactions for co-diagnosed substance use or substance exposure at birth. Model 3 additionally controls for other sociodemographic and pregnancy-related covariates. All analyses were conducted using Stata SE, version 13 (StataCorp LLC, College Station, TX, USA).

## 3. Results

### 3.1. Characteristics of Births with a Maternal Mental Health Disorder

As shown in Table 1, among the 551,232 infants born in 2006 and linked to a maternal hospital discharge record, 2.8% (*n* = 15,516) were born to mothers with a documented MH disorder in their medical records. Overall, the majority (93.5%) of mothers with a MH disorder received the general diagnosis of 648.4 or “Mental disorders complicating pregnancy, childbirth, or the puerperium” (herein referred to as MH Disorder at Delivery). Births with a maternal MH disorder were associated with younger maternal age at first birth, a higher percentage of White mothers (49.2% vs. 27.0%), more coverage by public health insurance (56.3% vs. 48.2%), and higher rates of missing paternity on the birth certificate (27.5% vs. 8.7%). The percentage of mothers who received prenatal care during the first trimester was lower among mothers with a MH disorder (70.7% vs. 85.7%). Notably, 41.3% of infants with mothers who had a MH disorder had documented maternal substance abuse versus less than 0.5% of infants born to mothers without a diagnosed MH disorder.

Differences emerged among mothers with specific diagnoses of psychotic, mood, or anxiety disorders at birth compared with the diagnosis of MH Disorder at Delivery. Mothers with at least one of these specific diagnoses tended to be older at the time of their first birth than mothers with a MH Disorder at Delivery diagnosis, with roughly 40% aged 30 years or older. Among mothers with diagnosed psychotic disorders, Black mothers were disproportionately represented. Nearly a quarter of mothers with a psychotic disorder were Black (23.4%) versus only 5.4% of mothers without a MH disorder. White mothers were overrepresented among those classified with mood and anxiety disorders. Public insurance covered 80.7% of births in which there was a diagnosed psychotic disorder, versus only 40.5% and 29.9% of those with mood and anxiety disorders, respectively. Nearly 50% of births to mothers with psychotic disorders were missing paternity, and one third had started prenatal care only after the first trimester or not at all. Underscoring the distinct profiles of mothers with specific MH disorder diagnoses versus a general diagnosis at delivery is the percentage of mothers with co-diagnosed substance exposure, which ranged from 5.4% (anxiety disorder) to 24.3% (psychotic disorder) for specific diagnoses compared to 44.0% for a MH Disorder at Delivery.

### 3.2. Maltreatment Reports to Child Protective Services

**Figure 1:**
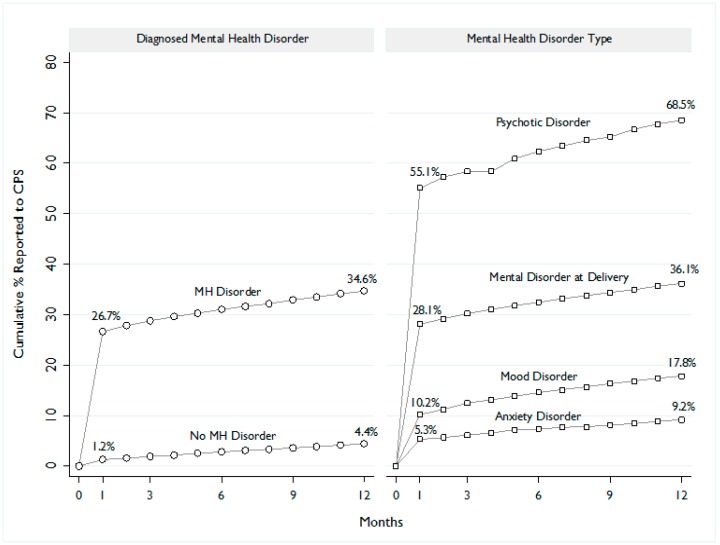
Cumulative percentage of infants reported to CPS during the first 12 months of life based on the diagnosis and type of maternal MH disorder.

Figure 1 depicts the cumulative percentage of infants reported to CPS during the first 12 months of life based on the diagnosis and type of maternal MH disorder. Overall, more than one third (34.6%) of infants born to mothers with a MH disorder were reported before one year of age, and a majority of those initial reports to CPS were made within the infant’s first month of life (77.2%). In contrast, among children born to mothers without a MH disorder, 4.4% were reported to CPS during the first year of life.

Among children born to mothers with a documented psychotic disorder, 68.5% were reported to CPS during infancy. However, the cumulative percentage reported to CPS was much lower among mother-child dyads in which anxiety or mood disorders were diagnosed (17.8% and 9.2%, respectively), yet these values were still twice as high as the percentage of those without any documented MH disorder (4.4%).

### 3.3. Association between Maternal Mental Health Disorders and Maltreatment Reporting

Table 2 presents findings from three different models examining the unadjusted and adjusted association between maternal MH disorders (with and without co-documented substance exposure) and the likelihood of a maltreatment report to CPS during infancy. As shown in Model 1, children born to mothers with any MH disorder were reported to CPS at nearly 8 times the rate of those whose mothers were without a MH disorder (RR: 7.82; 95% CI: 7.63, 8.02). The increased risk of reporting was most pronounced among births with a specific diagnosis of a psychotic disorder (RR: 13.10; 95% CI: 12.09, 14.19), although significant differences (*p* < 0.001) also emerged for births in which the specific diagnosis was for a mood or anxiety disorder.

Model 2 additionally examined the influence of co-documented substance exposure on CPS reporting. The combined effect of a maternal MH disorder and substance exposure was associated with a rate of CPS reporting 5 to 21 times that of other infants. For example, among mothers with a MH Disorder at Delivery diagnosis but no co-occurring substance abuse disorder, the RR was 3.16 (95% CI: 3.46, 3.84). The RR increased to 16.36 (95% CI: 15.54, 17.22) among mothers with a MH Disorder at Delivery diagnosis and comorbid substance use.

Finally, in Model 3, attempts to better isolate the relationship between maternal MH disorders and CPS reporting by adjusting for the covariates presented in Table 1 were made. After controlling for these covariates, the children of mothers with any maternal MH disorder but no a substance use disorder were still reported to CPS more during the first year of life at more than twice the rate of children born to mothers without a diagnosed MH disorder (RR: 2.60; 95% CI: 2.47, 2.73). Among infants born to mothers with both a maternal MH disorder and substance exposure, the risk remained even greater (RR: 5.69; 95% CI: 5.51, 5.87), albeit notably lower in the context of covariate adjustments.

## 4. Discussion

In the present study, linked administrative records were used to examine the relationship between maternal MH disorders documented at birth and CPS reporting during infancy. Several findings emerged that highlight the potential to not only use administrative records to monitor population-level dynamics, but also generate new knowledge concerning the relationship between maternal MH and CPS involvement.

First, this study provides a statewide estimate of the prevalence of infants born to mothers with MH disorders in the 2006 birth cohort. The percentage of MH disorders documented in the hospital delivery records of mothers in California increased from 1.7% in 1995 [17] to 2.8% a decade later, whereas the proportion of documented co-occurring MH and substance use disorders decreased. Additional research is needed to determine whether substance abuse has, indeed, decreased among mothers with a MH diagnosis or whether these findings reflect changes that occurred in documentation practices over the 10-year period. Whereas these estimates may be imperfect measures of the true prevalence of maternal MH disorders at birth, they underscore the potential for using hospital discharge data for population-level health surveillance. By focusing on mothers in which MH disorders were known to service providers at delivery, this analysis has implications for targeted prevention and early intervention services. Future studies would benefit from the inclusion of postpartum MH diagnoses in the data, which were not available for the present study.

Second, the present study produced a first-ever estimate of the occurrence of alleged maltreatment among infants born to mothers with MH disorders among both publicly and privately insured mothers in California. Statewide, one third of infants born to mothers with a MH disorder were reported to CPS by 12 months of age compared to fewer than one in every 20 infant born to a mother without a documented MH disorder. There were notable differences in the likelihood of CPS reporting by the specific MH disorder diagnosed. Overall, two thirds of infants born to mothers with a psychotic disorder were reported to CPS, which represents a rate more than 4 times that of infants born to mothers without a MH disorder after controlling for other risk factors at birth. Infants born to mothers with a mood disorder were more than twice as likely to have a CPS report, and infants born to mothers with an anxiety disorder experienced a small, but significant increase relative to infants of mothers without a MH disorder. Parental MH information is not always made available to child welfare social workers, and when provided, it is not systematically documented within CPS record systems when available. These findings provide useful estimates for understanding the scope of MH supports needed to service CPS-involved families. Further, the variations observed among CPS contact across the different types of MH disorders have implications for the development of child safety and risk-assessment tools and case-related decision making [28].

Third, for a majority of infants born to mothers with MH diagnoses who experienced a CPS report, the first report was made during the first month of life. This early reporting pattern indicates that those making a report to CPS were concerned about the mother’s capacity to care for her infant shortly after delivery and responded proactively. One could argue that high rates of CPS reporting immediately following childbirth may be more indicative of preconceptions about mental illness than of actual risk to a child’s safety. However, results from the present study also indicate that mothers with MH disorders who were not reported to CPS immediately following childbirth still had a comparatively higher occurrence of CPS reports by the infant’s first birthday compared with infants born to mothers without a MH diagnosis. This finding supports earlier work suggesting that maternal mental illness may have a lasting impact on an infant’s safety and wellbeing [14]. The decision to use administrative records for population surveillance undoubtedly requires ethical consideration; however, despite the risks, the present findings have the potential to enable better-informed decision making, communication, and collaboration between the MH and child welfare fields.

Finally, the present study highlights the risk associated with co-occurring MH and substance abuse diagnoses. Previous literature has documented an association among child maltreatment, parental mental illness [8], and substance abuse [29,30]. Findings from this study indicate that comorbid substance use amplifies the likelihood of CPS contact among mothers with MH diagnoses and that substance use may impact mothers differently based on their specific MH diagnosis type, even after controlling for covariates. This finding suggests that whereas a MH diagnosis may indicate a heightened level of risk for an infant, information about maternal substance use will provide critical information for understanding the risk level of child maltreatment. Mothers who are known to have co-occurring MH and substance abuse diagnoses are in need of enhanced services to prevent CPS involvement. Findings from the present study highlight the importance of collaboration among obstetrics, pediatric, MH, drug treatment, and child welfare service providers.

There are at least four limitations to this study. First, ICD codes are an imperfect measure of maternal mental illness, as all administrative data analyses are limited by errors in data entry [31], suggesting that ICD codes undoubtedly provide an undercount of the true occurrence of maternal MH and substance use disorders [31]. Yet, these codes offer a standardized tracking method that can be used to evaluate changes over time and are available for full birth cohorts in the hospital setting. Future research would benefit from the inclusion of ICD codes documented by outpatient service providers. Second, there may be surveillance bias in screening, diagnosing, and documenting individuals who receive diagnoses and are reported to CPS; for example, one earlier study found that sociodemographic risk factors increased the likelihood that a new mother’s MH and substance use symptoms and diagnoses would be documented [16]. Third, whereas the MH Disorder at Delivery code fell within the parameters of MH problems defined for this study and was included in earlier California analyses [16,17], this code appears to be used as a catchall diagnostic category and may, therefore, document MH disorders that would more appropriately be classified as psychotic, mood, or anxiety disorders. Future research regarding maternal MH disorders would benefit from a more in-depth study of the use and significance of the mental disorders at delivery category. Fourth, the year and geographic location in which the data were collected limit the generalizability of this study’s findings.

## 5. Conclusions

Infants born to mothers with a diagnosed MH disorder at the time of birth have a heightened likelihood of experiencing early CPS involvement, particularly in infants born to mothers with psychotic disorders or with a comorbid substance use disorder. CPS reports are most likely to occur during the child’s first month of life, making this a critical window for intervention. Early identification of infants born to mothers with MH disorders and co-occurring substance abuse should be viewed as an opportunity to provide parents with integrated support services early to prevent the occurrence of child maltreatment. Administrative records provide a cost-effective method for developing population-level data to inform policy development and improve service delivery.

## Figures and Tables

**Table 1:** Infants born in 2006 and linked to a maternal medical record.

Variable	No Mental Health Disorder		Any Mental Health Disorder		χ^2^	Psychotic Disorders		Mood Disorders		Anxiety Disorders		MH Disorder at Delivery	
	(*N* = 535,716)		(*N* = 15,516)			(*N* = 276)		(*N* = 5290)		(*N* = 1623)		(*N* = 14,508)	
	*n*	%	*n*	%	*p*-Value		*n*	%	*n*	%	*n*	%	*n*
Age at First Ever Birth					<0.001								
≤19 years	106,650	19.9	3417	22		43	15.6	897	17.0	186	11.5	3263	22.5
20–24 years	125,594	23.4	3913	25.2		59	21.5	1111	21.0	329	20.3	3701	25.5
25–29 years	130,333	24.3	3541	22.8		64	23.3	1213	22.9	427	26.3	3283	22.6
30+ years	173,106	32.3	4633	29.9		109	39.6	2068	39.1	681	42.0	4249	29.3
Race/Ethnicity					<0.001								
White	144,609	27.0	7630	49.2		107	38.8	2869	54.2	878	54.1	7160	49.4
Black	29,104	5.4	1781	11.5		66	23.9	383	7.2	81	5.0	1715	11.8
Hispanic	286,036	53.4	4911	31.7		69	25.0	1656	31.3	522	32.2	4526	31.2
Other/Missing	75,967	14.2	1194	7.7		34	12.3	382	7.2	142	8.7	1107	7.6
Insurance Type					<0.001								
Private	276,853	51.8	6730	43.7		53	19.3	3140	59.5	1137	70.1	6094	42.4
Public	257,953	48.2	8665	56.3		221	80.7	2138	40.5	484	29.9	8294	57.6
Paternity					<0.001								
Established	488,910	91.3	11,251	72.5		144	52.2	4393	83.0	1445	89.0	10,396	71.7
Missing	46,806	8.7	4265	27.5		132	47.8	897	17.0	178	11.0	4112	28.3
Prenatal Care					<0.001								
1st Trimester	459,200	85.7	10,974	70.7		185	67.0	4564	86.3	1447	89.2	10,095	69.6
2nd Trimester	58,806	11.0	2722	17.5		52	18.8	524	9.9	134	8.3	2633	18.1
3rd Trimester/No Care	17,710	3.3	1820	11.7		39	14.1	202	3.8	42	2.6	1780	12.3
Parity					<0.001								
First Birth	209,026	39.0	5474	35.3		102	37.0	2037	38.5	676	41.7	5029	34.7
Non-First Birth	326,690	61.0	10,042	64.7		174	63.0	3253	61.5	947	58.3	9479	65.3
Substance Abuse					<0.001								
No	534,132	99.7	9106	58.7		209	75.7	4730	89.4	1535	94.6	8128	56.0
Yes	1584	0.3	6410	41.3		67	24.3	560	10.6	88	5.4	6380	44.0

**Table 2:** Association between maternal MH disorders (with and without co-documented substance exposure) and the likelihood of a maltreatment report to CPS during infancy.

Variable	Model 1		Model 2		Model 3	
	Risk Ratio	(95% CI)	Risk Ratio	(95% CI)	Risk Ratio	(95% CI)
Any Mental Health Disorder	7.82	7.63, 8.02	3.53	3.37, 3.71	2.60	2.47, 2.73
Any Mental Health Disorder + Substance	--	--	13.91	13.59, 14.23	5.69	5.51, 5.87
Psychotic Disorder	13.10	12.09, 14.19	12.11	10.92, 13.42	4.60	3.96, 5.34
Psychotic Disorder + Substance	--	--	20.97	18.10, 24.29	5.67	4.50, 7.14
Mood Disorder	3.47	3.27, 3.68	2.56	2.38, 2.76	2.22	2.07, 2.39
Mood Disorder + Substance	--	--	13.22	10.88, 16.07	4.61	4.18, 5.09
Anxiety Disorder	1.74	1.49, 2.03	1.28	1.07, 1.55	1.43	1.20, 1.70
Anxiety Disorder + Substance	--	--	12.24	7.14, 21.02	3.87	3.01, 5.00
Mental Disorder at Delivery	8.13	7.93, 8.34	3.65	3.46, 3.84	2.60	2.47, 2.74
Mental Disorder at Delivery + Substance	--	--	16.36	15.54, 17.22	5.65	5.47, 5.83

Model 1 displays the risk of being reported during infancy among children born to mothers with a medically documented MH condition compared with children whose mother was not documented to have a MH condition. Model 2 first compares children born to mothers with a MH diagnosis but no substance abuse diagnosis relative to children whose mothers did not have a MH condition and second shows the risk for children born to mothers with a comorbid MH and substance abuse condition documented relative to children born to moms with no MH condition. Model 3 builds upon Model 2 with adjustments for other risk factors present at birth.

## Appendix A

**Table A1:** ICD-9-CM Codes.

Mental Health Disorders	Clinical Classification Software #	ICD-9-CM Codes
Psychotic Disorders	659	29381 29382 29500 29501 29502 29503 29504 29505 29510 29511 29512 29513 29514 29515 29520 29521 29522 29523 29524 29525 29530 29531 29532 29533 29534 29535 29540 29541 29542 29543 29544 29545 29550 29551 29552 29553 29554 29555 29560 29561 29562 29563 29564 29565 29570 29571 29572 29573 29574 29575 29580 29581 29582 29583 29584 29585 29590 29591 29592 29593 29594 29595 2970 2971 2972 2973 2978 2979 2980 2981 2982 2983 2984 2988 2989
Mood Disorders	657	29383 29600 29601 29602 29603 29604 29605 29606 29610 29611 29612 29613 29614 29615 29616 29620 29621 29622 29623 29624 29625 29626 29630 29631 29632 29633 29634 29635 29636 29640 29641 29642 29643 29644 29645 29646 29650 29651 29652 29653 29654 29655 29656 29660 29661 29662 29663 29664 29665 29666 2967 29680 29681 29682 29689 29690 29699 3004
Anxiety Disorders	651	29384 30000 30001 30002 30009 30010 30020 30021 30022 30023 30029 3003 3005 30089 3009 3080 3081 3082 3083 3084 3089 30981 3130 3131 31321 31322
Mental Disorders at Delivery	648	64841 64842
Other Mental Health Disorders		
Adjustment Disorders	650	3090 3091 30922 30923 30924 30928 30929 3093 3094 30982 30983 30989 3099
Attention Deficit, Conduct, and Disruptive Behavior Disorders	652	31200 31201 31202 31203 31210 31211 31212 31213 31220 31221 31222 31223 3124 31281 31282 31289 3129 31381 31400 31401 3141 3142 3148 3149
Delirium, Dementia, and other Cognitive Disorders	653	2900 29010 29011 29012 29013 29020 29021 2903 29040 29041 29042 29043 2908 2909 2930 2931 2940 2941 29410 29411 29420 29421 2948 2949 3100
Developmental Disorders	654	3070 3079 31500 31501 31502 31509 3151 3152 31531 31532 31534 31535 31539 3154 3155 3158 3159 317 3180 3181 3182 319 V400 V401
Impulse Control Disorders	656	31230 31231 31232 31233 31234 31235 31239
Personality Disorders	658	3010 30110 30111 30112 30113 30120 30121 30122 3013 3014 30150 30151 30159 3016 3017 30181 30182 30183 30184 30189 3019
Miscellaneous Disorders	670	29389 2939 30011 30012 30013 30014 30015 30016 30019 3006 3007 30081 30082 3021 3022 3023 3024 30250 30251 30252 30253 3026 30270 30271 30272 30273 30274 30275 30276 30279 30281 30282 30283 30284 30285 30289 3029 3060 3061 3062 3063 3064 30650 30651 30652 30653 30659 3066 3067 3068 3069 3071 30740 30741 30742 30743 30744 30745 30746 30747 30748 30749 30750 30751 30752 30753 30754 30759 30780 30781 30789 3101 316 64840 64841 64842 64843 64844 V402 V4031 V4039 V409 V673
Alcohol-related Substance Use Disorders	660	2910 2911 2912 2913 2914 2915 29181 29182 29189 2919 30300 30301 30302 30303 30390 30391 30392 30393 30500 76071 9800
Drug-related Substance Use Disorders	661	2920 29211 29212 2922 29281 29282 29283 29284 29285 29289 2929 30400 30401 30402 30403 30410 30411 30412 30413 30420 30421 30422 30423 30430 30431 30432 30433 30440 30441 30442 30443 30450 30451 30452 30453 30460 30461 30462 30463 30470 30471 30472 30473 30480 30481 30482 30483 30490 30491 30492 30493 30520 30521 30522 30523 30530 30531 30532 30533 30540 30541 30542 30543 30550 30551 30552 30553 30560 30561 30562 30563 30570 30571 30572 30573 30580 30581 30582 30583 30590 30591 30592 30593 64830 64831 64832 64833 64834 65550 65551 65553 76072 76073 76075 7795 7795 96500 96501 96502 96509 V6542

## References

[1] Foster K., O’Brien L., Korhonen T. (2012). Developing resilient children and families when parents have mental illness: A family focused approach. Int. J. Ment. Health Nurs..

[2] Jablensky A.V., Morgan V., Zubrick S.R., Bower C., Yellachich L.A. (2005). Pregnancy, delivery, and neonatal complications in a population cohort of women with schizophrenia and major affective disorders. Am. J. Psychiatry.

[3] Hoirisch-Clapauch S., Brenner B., Nardi A.E. (2015). Adverse obstetric and neonatal outcomes in women with mental disorders. Thromb. Res..

[4] Accortt E.E., Cheadle A.C., Schetter C.D. (2015). Prenatal depression and adverse birth outcomes: An updated systematic review. Matern. Child Health J..

[5] Bodén R., Lundgren M., Brandt L., Reutfors J., Andersen M., Kieler H. (2012). Risks of adverse pregnancy and birth outcomes in women treated or not treated with mood stabilisers for bipolar disorder: Population based cohort study. BMJ.

[6] Lee H.C., Lin H.C. (2010). Maternal bipolar disorder increased low birthweight and preterm births: A nationwide population-based study. J. Affect. Disord..

[7] Dietz P.M., Williams S.B., Callaghan W.M., Bachman D.J., Whitlock E.P., Hornbrook M.C. (2007). Clinically identified maternal depression before, during, and after pregnancies ending in live births. Am. J. Psychiatry.

[8] Brockington I.A., Chandra P., Dubowitz H., Jones D., Moussa S., Nakku J., Ferre I.Q. (2011). WPA guidance on the protection and promotion of mental health in children of persons with severe mental disorders. World Psychiatry.

[9] Lewin L., Abdrbo A. (2009). Mothers with self-reported Axis I diagnoses and child protection. Arch. Psychiatr. Nurs..

[10] Mullick M., Miller L.J., Jacobsen T. (2001). Insight into mental illness and child maltreatment risk among mothers with major psychiatric disorders. Psychiatr. Serv..

[11] Brady K.T., Sinha R. (2005). Co-occurring mental and substance use disorders: The neurobiological effects of chronic stress. Am. J. Psychiatry.

[12] Kohl P.L., Jonson-Reid M., Drake B. (2011). Maternal mental illness and the safety and stability of maltreated children. Child Abuse Negl..

[13] Ackerson B.J. (2003). Parents with serious and persistent mental illness: Issues in assessment and services. Soc. Work.

[14] Conroy S., Marks M.N., Schacht R., Davies H.A., Moran P. (2010). The impact of maternal depression and personality disorder on early infant care. Soc. Psychiatry Psychiatr. Epidemiol..

[15] Park J.M., Solomon P., Mandell D.S. (2006). Involvement in the child welfare system among mothers with serious mental illness. Psychiatr. Serv..

[16] Kelly R.H., Zatzick D.F., Anders T.F. (2001). The detection and treatment of psychiatric disorders and substance use among pregnant women cared for in obstetrics. Am. J. Psychiatry.

[17] Kelly R.H., Danielsen B.H., Golding J.M., Anders T.F., Gilbert W.M., Zatzick D.F. (1999). Adequacy of prenatal care among women with psychiatric diagnoses giving birth in California in 1994 and 1995. Psychiatr. Serv..

[18] O’Donnell M., Maclean M.J., Sims S., Morgan V.A., Leonard H., Stanley F.J. (2015). Maternal mental health and risk of child protection involvement: Mental health diagnoses associated with increased risk. J. Epidemiol. Community Health.

[19] Herrchen B., Gould J.B., Nesbitt T.S. (1997). Vital statistics linked birth/infant death and hospital discharge record linkage for epidemiological studies. Comput. Biomed. Res..

[20] Prindle J.J., Hammond I., Putnam-Hornstein E. (2017). Prenatal Substance Exposure Diagnosed at Birth and Infant Involvement with Child Protective Services. Child Abuse Negl..

[21] Kohl P.L., Jonson-Reid M., Drake B. (2009). Time to leave substantiation behind: Findings from a national probability study. Child Maltreat..

[22] Hussey J.M., Marshall J.M., English D.J., Knight E.D., Lau A.S., Dubowitz H., Kotch J.B. (2005). Defining maltreatment according to substantiation: Distinction without a difference?. Child Abuse Negl..

[23] World Health Organization (1977). International Classification of Diseases: Manual of the International Classification of Diseases, Injuries, and Causes of Death.

[24] Da Silva A.A., Simões V.M., Barbieri M.A., Bettiol H., Lamy-Filho F., Coimbra L.C., Alves M.T. (2003). Young maternal age and preterm birth. Paediatr. Perinat. Epidemiol..

[25] Fall C.H., Sachdev H.S., Osmond C., Restrepo-Mendez M.C., Victora C., Martorell R., Stein A.D., Sinha S., Tandon N., Adair L. (2015). Association between maternal age at childbirth and child and adult outcomes in the offspring: A prospective study in five low-income and middle-income countries (COHORTS collaboration). Lancet Glob. Health.

[26] McCullagh P., Nelder J.A. (1989). Generalized Linear Models.

[27] UCLA Academic Technology Services: Statistical Consulting Group. Stata FAQ: How Can I Estimate Relative Risk Using GLM for Common Outcomes in Cohort Studies?. http://www.ats.ucla.edu/stat/stata/ado/analysis/.

[28] Johnson W. (2004). Effectiveness of California’s Child Welfare Structured Decision Making (SDM) Model: A Prospective Study of the Validity of the California Family Risk Assessment.

[29] Walsh C., MacMillan H., Jamieson E. (2002). The relationship between parental psychiatric disorder and child physical and sexual abuse: Findings from the Ontario Health Supplement. Child Abuse Negl..

[30] O’Donnell M., Nassar N., Leonard H., Hagan R., Mathews R., Patterson Y., Stanley F. (2009). Increasing prevalence of neonatal withdrawal syndrome: Population study of maternal factors and child protection involvement. Pediatrics.

[31] Drake B., Jonson-Reid M. (1999). Some thoughts on the increasing use of administrative data in child maltreatment research. Child Maltreat..

